# Signaling Pathways Induced by Leptin during Epithelial–Mesenchymal Transition in Breast Cancer

**DOI:** 10.3390/ijms19113493

**Published:** 2018-11-06

**Authors:** Monserrat Olea-Flores, Juan Carlos Juárez-Cruz, Miguel A. Mendoza-Catalán, Teresita Padilla-Benavides, Napoleón Navarro-Tito

**Affiliations:** 1 Laboratorio de Biología Celular del Cáncer, Facultad de Ciencias Químico Biológicas, Universidad Autónoma de Guerrero, Av. Lázaro Cárdenas s/n, Chilpancingo, GRO 39090, México; monse-olea@live.com.mx (M.O.-F.); jcjuarezc91@gmail.com (J.C.J.-C.); 2 Laboratorio de Biomedicina Molecular, Facultad de Ciencias Químico Biológicas, Universidad Autónoma de Guerrero, Av. Lázaro Cárdenas s/n, Chilpancingo, GRO 39090, México; mamendoza@uagro.mx; 3 Department of Biochemistry and Molecular Pharmacology, University of Massachusetts Medical School, 364 Plantation Street, Worcester, MA 01605, USA; Teresita.Padilla@umassmed.edu

**Keywords:** leptin, EMT, transcription factors, breast cancer

## Abstract

Leptin is an adipokine that is overexpressed in obese and overweight people. Interestingly, women with breast cancer present high levels of leptin and of its receptor ObR. Leptin plays an important role in breast cancer progression due to the biological processes it participates in, such as epithelial–mesenchymal transition (EMT). EMT consists of a series of orchestrated events in which cell–cell and cell–extracellular matrix interactions are altered and lead to the release of epithelial cells from the surrounding tissue. The cytoskeleton is also re-arranged, allowing the three-dimensional movement of epithelial cells into the extracellular matrix. This transition provides cells with the ability to migrate and invade adjacent or distal tissues, which is a classic feature of invasive or metastatic carcinoma cells. In recent years, the number of cases of breast cancer has increased, making this disease a public health problem worldwide and the leading cause of death due to cancer in women. In this review, we focus on recent advances that establish: (1) leptin as a risk factor for the development of breast cancer, and (2) leptin as an inducer of EMT, an event that promotes tumor progression.

## 1. Introduction

Leptin is a protein that plays an important role in regulating energy homeostasis and neuroendocrine and immune functions, in addition to glucose and lipid metabolism [1]. It is a pleiotropic molecule that participates in regulating immunity, inflammation, cell differentiation, and the proliferation of different types of cells, including mammary epithelial cells [2,3]. However, diverse studies have shown that leptin and its receptor, ObR, are overexpressed in patients with breast cancer [4]. In in vitro models, such as MCF7, MDA-MB-231, and SK-BR-3 breast cancer cells, leptin activates signaling pathways that promote proliferation, cell migration, invasion, and epithelial–mesenchymal transition (EMT) [5,6,7].

EMT is a process of trans-differentiation by which epithelial cells change to a mesenchymal phenotype [8]. This process is characterized by the loss of epithelial markers and the acquisition of mesenchymal markers; both molecular events contribute to tumor progression [9]. The regulation of EMT markers is due to the activation of some transcription factors (TF), such as Snail, Slug, Zeb, Twist, and β-catenin [6,10]. These TFs are involved in transcriptional repression of genes associated with the epithelial phenotype, such as E-cadherin, occludin, and claudins [10,11]. The repression of the expression of these molecules involved in cell adhesion, lead to the loss of cell junctions and apico-basolateral polarity [9]. Also, the trans-differentiation program is characterized by the expression of mesenchymal markers such as vimentin, N-cadherin, and matrix metalloproteases (MMPs) [12,13]. The EMT process has been classified according to three different biological contexts: (a) Type 1, essential for embryonic development; (b) Type 2, which is linked with wound healing and tissue regeneration; (c) Type 3, associated with tumor progression [14].

Breast cancer is a severe public health problem worldwide. The World Health Organization estimates 1.38 million new cases and 458,000 deaths reported every year, making this tumor the main cause of death by cancer in women [15]. Breast cancer is a heterogeneous disease that arises from the hyperplasia of the epithelial cells confined in ducts or lobes of the mammary gland [16]. This hyperplasia can progress to a ductal or lobular carcinoma in situ, where the EMT program can be activated, to provide tumor cells with properties that facilitate the invasion of adjacent or distal tissues [16]. One of the main risk factors for the development and progression of breast cancer is obesity, which is defined as an abnormal or excessive accumulation of fat in an individual that can be harmful [17]. Obesity is characterized by the accumulation of adipose tissue and an increase in the body mass index (BMI > 30) [14]. An important characteristic of obesity is the dysregulation of adipokine levels, such as leptin and adiponectin, secreted by the adipose tissue [18]. Importantly, a decrease in adiponectin and an increase in leptin levels favor the development and progression of breast cancer [14,19]. In this review, we focus on the molecular role of leptin as an inducer of EMT and its implications in breast cancer.

## 2. Leptin Signaling

Leptin is an adipokine with a molecular weight of 16 kDa, encoded by the *LEP* gene, located on chromosome 7q32.1 [20,21]. Leptin is secreted mainly by the adipose tissue [21] and, in a smaller proportion, by the placenta [22], stomach [23], fibroblasts [24], skeletal muscle [25], normal and tumor epithelial mammary tissue [26,27,28]. Leptin regulates the appetite through binding to the ObR receptor, which is located in neurons of the arcuate nucleus (ARC) [29]. Upon leptin binding, ObR activates the PI3K signal pathway to inhibit the expression of orexigenic neuropeptides such as neuropeptide Y (NPY) and induces the hyperpolarization of ARC neurons [30]. Leptin also depolarizes the hypothalamic proopiomelanocortin (POMC) neurons and activates anorexigenic neuropeptides such as POMC and CART (Cocaine- and Amphetamine-Regulated Transcript), to further regulate food intake [31,32].

**Figure 1:**
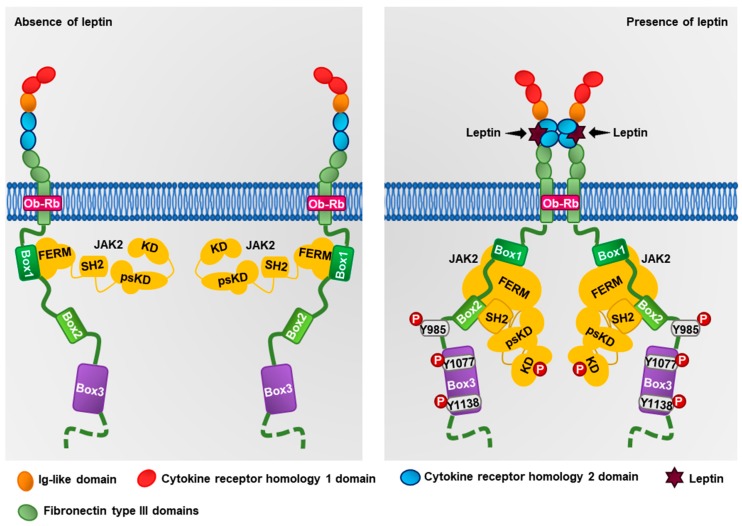
Structure of the ObRb receptor. The ObR is constituted by an extracellular N-terminus domain, a transmembranal domain, and a cytoplasmic C-terminus domain. In the absence of leptin, ObR is located in the plasma membrane as a monomer associated with inactive JAK. Upon leptin binding to ObRb, ObRb dimerizes, and the JAK kinase is autophosphorylated, favoring its activation. Once active, JAK2 phosphorylates tyrosine residues in ObR and activates downstream signaling pathways.

Leptin exerts its effects through its ObR receptor, which is encoded by the *LEPR* gene located on chromosome 1p31.3 [33]. The ObR receptor belongs to the family of class I cytokine receptors [25,26], and presents six isoforms generated by alternative splicing (ObRa–ObRf) [33]. Interestingly, only the ObRb isoform contains the intracellular motifs required for the activation of the signaling cascade mediated by JAK2, an ObRb-associated kinase [34,35]. Structurally, the extracellular region of the ObRb receptor is constituted by two cytokine homology regions (CHR), modulating the binding with leptin (Figure 1). However, only the CHR2 domain is necessary for the binding with leptin; both domains are separated by an immunoglobulin-like domain (IgD) [36]. The ObR also contains two or three domains of fibronectin type III which regulate their interaction with cell membranes [36]. In the intracellular region, the ObRb contains three boxes, a proline-rich region called box 1, essential for the binding of the FERM domain of JAK2, a region called box 2, which interacts with the SH2 domain of JAK2, and the box 3, containing Tyr1077 and Tyr1138, necessary residues for the activation of STA3 and STAT5 [37]. Structurally, JAK2 is constituted by a pseudokinase domain (psKD), a kinase domain (KD), an SH2 domain, and a FERM domain. The FERM and SH2 domains are responsible for regulating the interaction of JAK2 with box 1 and 2 of the ObRb receptor, whereas psKD and KD are responsible for regulating JAK2 kinase activity [38]. Leptin can bind to the CHR2 domain of ObRb, promoting trans-dimerization of two leptin–ObRb dimers [36,39]. This event induces changes in the intracellular region of the ObRb receptor, as well as a conformational change in JAK2, promoting its autophosphorylation and subsequent activation (Figure 1) [34,37,39]. JAK2 can phosphorylate multiple sites in the ObRb receptor, activating several signaling pathways. For instance, JAK2 phosphorylates Tyr1077 leading to the activation of STAT5, which translocates to the nucleus and regulates gene expression [40,41]. JAK2 also phosphorylates ObRb at Tyr1138, which is recognized by the SH2 domain of STAT3 [42]. Also, JAK2 phosphorylates STAT3 at Tyr705, inducing its dimerization and translocation to the nucleus where it regulates the expression of different proteins involved in cancer progression, such as cyclin D1, COX2, VEGF, and SOCS3, a negative regulator of leptin signaling [35,43]. Also, leptin induces the phosphorylation of the insulin receptor substrate 1 (IRS-1) through JAK2, triggering the activation of the PI3K–Akt pathway [7,44]. On the other hand, JAK2 phosphorylates Tyr985 of ObRb, allowing the anchoring of SHP2 protein [45,46], promoting the recruitment of Grb2 and the activation of the kinases ERK1/2 [47]. The mechanism of inhibition of SOCS3 occurs when it binds through its SH2 domain to p-Tyr985 of ObRb, preventing the activation of the MAPKs pathway, and, through its C-terminal domain,, recruits the ubiquitin-transferase system inducing the ubiquitination of JAK2 [48]. Another negative regulator is PTP1B, a phosphatase that participates in STAT3, STAT5 and JAK2 dephosphorylation, thus inhibiting leptin signaling [49,50]. Together, these signaling pathways regulate cancer-related processes such as proliferation, survival, EMT, cell migration, and invasion [51,52,53].

## 3. Epithelial–Mesenchymal Transition (EMT)

The EMT is a process involved in a pathophysiological condition in which epithelial cells acquire characteristics of mesenchymal cells [9]. EMT involves a modification of the classic epithelial phenotype and morphology to a fibroblastoid phenotype, as it favors an increase of cell migration, invasion, and resistance to anoikis and chemotherapy [54,55]. In the molecular context, cells undergo changes in gene expression, function, and/or activation of proteins involved in this transition [56,57,58]. EMT is characterized by the loss of the cell–cell junctions and the reorganization of the cytoskeleton, which results in the loss of apicobasal polarity and the decrease in the expression of canonical epithelial markers such as E-cadherin, cytokeratins, ZO-1 [59]. This is followed by a gain of mesenchymal markers such as N-cadherin, vimentin, fibronectin, α-SMA, as well as an increase in the expression and activation of the TFs that regulate EMT, such as Twist, Snail, Slug, ZEB, and β-catenin [57,60]. These TFs regulate the expression of genes that favors cell migration and invasive processes [60,61] and contribute to the disruption of cell junctions by transcriptionally repressing *CDH1*, *OCLN*, and *CLDN* [62,63,64].

## 4. EMT-Related Transcription Factors

A hallmark of EMT is the dysregulation of E-cadherin, occludin, claudins, and cytokeratins which leads to the loss of the apico-basolateral polarity, mediated by the TFs Snail, Slug, Zeb, and Twist [10], which are discussed below.

### 4.1. Snail

**Figure 2:**
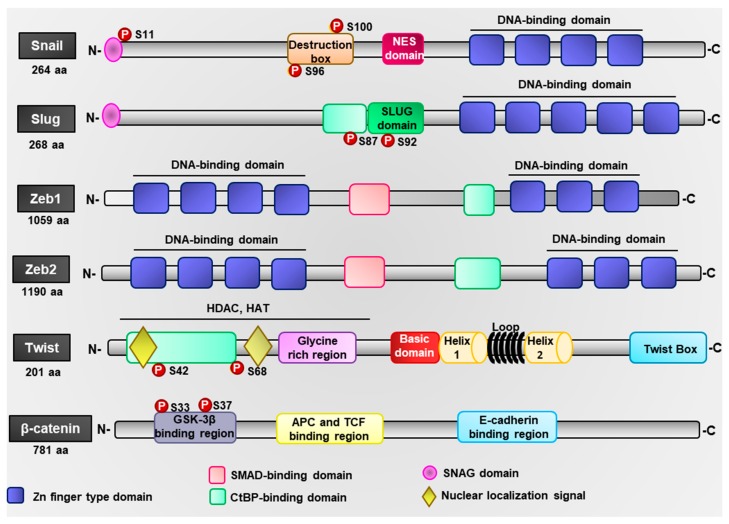
Schematic representation of leptin-induced transcription factors. Structural domains of the epithelial–mesenchymal transition (EMT)-related transcription factors (TFs), where the domains of DNA binding and interaction with other proteins are represented.

Snail (Snail1) and Slug (Snail2) are members of the Snail family [65]. The Snail gene (*SNAI1*) is located on chromosome 20q13.13 and codes for a 29 kDa protein (Figure 2) [66]. Snail is constituted by two β chains and two α helices and, in the C-terminal region, has four Zn-fingers domains responsible for the interaction with the consensus DNA sequence CACCTG [67]. This sequence is found in the E2 box of the promoter regions of genes such as *CDH1*, *MUC 1*, *KRT18*, *OCLN*, and *CLDN*, for which Snail functions as a repressor [11,67]. Snail presents an N-terminal SNAG domain, essential for transcriptional repression, and a nuclear export signal (NES) domain that regulates its nuclear export. In this way, Snail regulates the survival process through the repression of *CCND1* (cyclin D1), *CCND2* (cyclin D2), and *CDK4* (CDK4) [68]. In addition, Snail represses the expression of the tumor suppressor PTEN, preventing its interaction with p53 and leading to cell cycle blockage, further conferring resistance to cell death [68,69]. Snail also represses the expression of *CDH1* and *OCLN*, two genes involved in the formation of cell–cell junctions [11]. Snail further represses *SERPINB5* expression, which facilitates cell migration through the PI3K–Akt–Rac1 pathway [70]. The expression and activation of Snail are regulated by various signaling pathways including PI3K, MAPK, GSK-3β, and NF-κB [71,72,73]. ERK1/2 signaling regulates the activity of NF-κB, which controls the transcription of Snail [74,75,76]. Similarly, PI3K-mediated signaling promotes the activation of Akt, inducing the upregulation of NF-κB and increasing the transcriptional activation of Snail [77]. Furthermore, PI3K promotes the activation of PAK1, which phosphorylates Snail at Ser246, promoting its nuclear translocation and the control of the expression of EMT-related genes [78]. Additionally, SUMOylation of Snail at Lys234 stabilizes and promotes its translocation to the nucleus, allowing its interaction with c-Jun to regulate gene expression [79]. Snail phosphorylation at Ser11 by PKA and Ser92 by CK2 regulates the transcriptional repression of *CDH1* and *CLDN1* through the recruitment of the transcriptional repressor mSin3A and histone deacetylases (HDACs), responsible for decreasing the acetylation at H3 and H4 of *CDH1* promoter [80,81]. Snail also recognizes the TCACA conserved sequence on the *MMP9* gene promoter region and forms a complex with Early Growth Response proteins EGR/Sp1 to promote the transcription of this MMP [82]. This conserved DNA sequence is also recognized by Snail in the Zinc Finger E-Box Binding Homeobox 1 (*ZEB1*) promoter to regulate its expression [12]. However, in the nucleus, GSK3β phosphorylates Snail at Ser104 and Ser107 that are close to the NES sequence of Snail, inducing its nuclear export; then, in the cytoplasm, Snail phosphorylation at Ser96 and Ser100 by GSK3β inactivates it functionally, sending it to proteasome degradation through the ubiquitin ligase β-Trcp [83,84].

### 4.2. Slug

The Slug gene (*SNAI2*) is located on chromosome 8q11.2, and encodes a 28 kDa protein, formed by two β chains and two α helices, and containing five Zn-finger domains in the C-terminal domain (Figure 2) [85]. These domains recognize the consensus DNA sequence CAGGTG located in the E2 box of the *CDH1* promoter. Slug presents a SNAG and a SLUG domain which recruit transcriptional co-repressors such as CtBP-1, which in turn recruit HDAC to the promoters of target genes [86,87]. Slug is phosphorylated by ERK1/2 at Ser100 to promote its nuclear localization and at Ser87 to regulate its transcriptional activity [88]. Also, the SUMOylation of Slug at Lys192 increases its stability and promotes its ability to suppress the expression of E-cadherin [79]. Slug is degraded through the p21–p53–Mdm2 complex via ubiquitination [86,89,90] and through phosphorylation by GSK3β at Ser92 via proteasome degradation [88].

The overexpression of Slug correlated with metastasis, invasion, and decreased survival in patients with breast, gastric, lung, and ovarian cancer [91]. In vitro studies have shown that the ectopic expression of Slug in MCF10A cells induces a morphological change from an epithelial phenotype to a more elongated mesenchymal phenotype [92]. Slug induces a decrease in the levels of E-cadherin and β-catenin and an increase of vimentin levels [93]. Slug phosphorylation at Ser87 is associated with the overexpression of vimentin [92], which correlates with an increase in the migration capacity of MCF10A cells [94]. Furthermore, the expression of Slug correlates with the repression of *BRCA2* in breast cancer [87].

### 4.3. Zeb

The Zeb family of TFs has two members, Zeb1 (*TCF8*) and Zeb2 (*SiP1*). Zeb1 is encoded by the *ZEB1* gene located on chromosome 10p11.22, while Zeb2 is encoded by the *ZEB2* gene located on chromosome 2q22.3. Both proteins are characterized by two clustered Zn fingers separated by a central homeodomain (Figure 2) [95,96]. Zeb interacts with the DNA consensus sequences CACCT/G found in the E-box located in the promoter regions of targets genes, through the Zn-finger domain [97,98]. Similarly, Zeb interacts with transcriptional repressors through the sequence PXDLS [11,98]. Common interactors of Zeb are CtBP, HDAC, methyltransferases, polycomb complex, and coREST. In this way, Zeb inhibits the transcription of genes involved in the epithelial phenotype [95,99]. In addition, Zeb can be activated via Ras–ERK2–Fra1 signaling, NF-КB, and JAK–STAT3 [100,101,102].

Zeb interacts with the transcriptional modulators Smad1, 2, and 3. The Zeb1–Smad3–p300 complex can interact with Smad7 and displace HDAC1 to allow transcriptional activation [103]. However, when the Zeb2–Smad3 complex is formed, it binds to CtBP to repress the transcription of genes such as *CDH1* [98]. The promoter of Zeb presents four E-boxes, which are recognized by Slug and favor the transcription of Zeb [104]. The levels of Zeb are positively regulated by the TF Snail through the transcriptional repression of miR-200, a negative regulator of Zeb; in addition, Twist and Ets1 bind to the Zeb promoter, inducing its expression [105]. A mechanism of negative regulation of the transcriptional repression activity of Zeb is through SUMOylation at Lys 391 and 866; this event is mediated by the PC2 protein of the polycomb complex, which acts as a SUMO E3 ligase [79,106].

### 4.4. Twist

The Twist gene (*TWIST1*) is located on chromosome 7p21.1 and encodes a 28 kDa protein that presents two α-helices separated by a loop. Twist N-terminal region presents an HLH domain, that mediates specific DNA binding and contains two amphipathic helices that act as dimerization domains (Figure 2) [107,108]. These heterodimers recognize the consensus DNA sequence CANNTG in the promoter regions of target genes [108,109]. Also, the N-terminal region of Twist includes the nuclear localization signals, which spans from amino acids 37 to 40 and 73 to 77 [110]. Moreover, the phosphorylation of Ser42 and Ser68 by Akt2 and ERK1/2, respectively, also induces its translocation to the nucleus [110,111,112]. Phosphorylation of Thr121 and Ser123 by Akt1 in the HLH domain induces Twist degradation via ubiquitination [113,114]. Twist C-terminal domain contains the Twist-box domain that has a dual function, acting as a transcriptional activator or repressor [108]. Twist activity can be epigenetically regulated through its interaction with histone acetyltransferases and HDACs, which induce histone modifications and repress gene expression [115]. The overexpression of *Twist* in the human adenocarcinoma cell line MCF7 induces a change from an epithelial to a mesenchymal phenotype, accompanied by an increase in the synthesis of the angiogenic vascular endothelial growth factor (VEGF) [116]. Twist also promotes the expression of Snail and induces cell migration and invasion of MCF7 cells [117]. Furthermore, overexpression of Twist in the MCF7 and MCF10A cell lines promotes a breast cancer stem cell phenotype [117].

Twist and BMI1 TFs may suppress the expression of let-7i, a tumor suppressor miRNA, favoring the motility of mesenchymal cells and invasiveness to local and distant sites, and contributes to the maintenance of stem-cell-like properties [118].

### 4.5. β-Catenin

Another transcription factor involved in EMT is β-catenin, which is part of the transmembrane adherent junctions complex (AJs) and interacts with E-cadherin [119]. The loss of E-cadherin from the basolateral membrane is associated with a release of β-catenin to the cytosol and to the activation of the canonical Wnt pathway [120,121,122]. Wnt promotes cell growth, survival, and maintenance of stemness through the β-catenin–TCF3 complex by inhibiting the pluripotency of the factors Oct3/4, Sox2, and Nanog, thus maintaining the self-renewal capacity of cancer stem cells [120,121,122,123]. When AJs are lost, β-catenin can be phosphorylated at Ser33 and Ser37 by GSK3β and binds to the ubiquitin ligase β-Trcp, inducing its degradation via ubiquitination [124]. However, Akt and PI3K can phosphorylate GSK3β at Ser9 and prevent the formation of the subsequent destruction complex LKB1–APC–Axin, thus avoiding β-catenin degradation [6]. JNK2 also phosphorylates β-catenin at Ser191 and Ser605, promoting its nuclear translocation to regulate the expression of EMT-associated genes [125]. Overexpression of Twist, Snail, and Slug also promotes the nuclear localization of β-catenin [93,121]. In the nucleus, β-catenin constitutes a complex with TCF and LEF, which recognize the consensus DNA sequence T/A-CAAAG located in the HMG boxes of the promoter regions of EMT-associated genes [126]. Importantly, the promoter of Snail also presents this sequence, suggesting that it may be regulated the β-catenin–TCF–LEF complex as well [93,121]. The C-terminal domain of β-catenin may also interact with CBP–p300 and the chromatin remodeling enzyme BRG1, member of the SWI–SNF complex, allowing the transcription of c-Myc, Cyclin D1, c-Jun, fra-1 [122,127].

## 5. Expression of Leptin and ObR in Breast Cancer

### 5.1. Studies in Humans

Diverse studies in humans have reported that the levels of leptin in the serum of obese and overweight individuals are increased compared to subjects with normal weight [2,4]. In healthy individuals with normal weight, the concentration of leptin in the bloodstream is about 5–20 ng/mL [128,129,130,131], while, in a patient with breast cancer, the leptin levels reach up to 100 ng/mL [132,133]. Moreover, overexpression of leptin and its ObR receptor are associated with early stages of carcinogenesis [134]. For instance, patients with in situ ductal carcinoma present increased expression of the ObR receptor, compared to patients with invasive carcinoma [134]. The overexpression of leptin and the ObR receptor also promotes the progression of breast cancer. In line with this idea, Garofalo et al. showed that both leptin and ObR are increased in primary tumors and lymph node metastases of breast cancer [135]. Hosney et al. observed that leptin was significantly overexpressed in obese patients compared with overweight patients and healthy donors by 3.1-fold and 8.3-fold, respectively [26].

However, not only the overexpression of leptin and its receptor favors tumor progression, but also polymorphisms (variation in specific DNA sequences) of leptin or its receptor genes are considered potential mechanisms of enhanced susceptibility to develop breast cancer [136]. In the polymorphism of *LEP* G2548A, the associated allele A is the risk factor for the development of breast cancer, while, in the *LEPR* Q223R polymorphism, the R allele is linked to the development of the disease. Likewise, the A allele of *LEP* G2548A is associated with the size of the tumor [137]. Interestingly, the *LEP* G2548A polymorphism is related to variations in the levels of leptin in serum; however, this polymorphism was not related to the susceptibility to develop breast cancer [138,139]. On the other hand, the *LEPR* Q223R polymorphism, involved in receptor functionality, was shown to decrease the risk of developing breast cancer in Asian women but not in Caucasian women [138]. A study of Iranian women with breast cancer showed a higher frequency of breast cancer development associated with the *LEP* G2548A polymorphism of the A allele, as compared to the control group; interestingly, the polymorphism of the G allele conferred a protective phenotype [140]. Moreover, post-menopausal and pre-menopausal Mexican women appeared susceptible to develop breast cancer if the *LEPR* Q223R or the *LEP* G2548A polymorphisms, respectively, were present [141]. Egyptian patients with breast cancer frequently present the AA genotype of *LEP* G2548A compared to healthy women, while the *LEPR* Q223R polymorphism is associated with the development of breast cancer [142].

### 5.2. In Vitro Models

In vitro models have been used extensively to elucidate the mechanisms of leptin activation in biological processes associated with breast cancer progression. Established cell lines, such as MCF10A [143,144], MCF7 [143,144,145], T47D [143,144,145], and MDA-MB-231 [143,144,145], express both the long and the short isoforms of the ObR receptor. Elevated mRNA levels of the ObR receptor were observed in MCF10A [143,144], MDA-MB-231 [143,144,145], and MCF7 cells [143,144,145]. Leptin promotes the proliferation of MCF10A [143,144], MCF7 [143,144,145], T47D [143,144,145], Leal-10 [146], and MDA-MB-231 cells in culture [144,145,146,147,148]. Moreover, leptin induces cell migration and invasion of MCF7 [6,145], Leal-10 [146], and T47D cells [145] and decreased apoptosis in MCF7 [145] and ZR-75-1 cells [149]. Consistently, chronic treatment with leptin induces an increase in the population of cancer stem cells in the MDA-MB-231 cultured model [6,143,145,146]. Leptin also induces the expression of TFs associated with the maintenance of the cancer stem cell phenotype, such as NANOG, SOX2, and OCT4 in a STAT3-dependent manner, promoting a more aggressive phenotype of cancer cells [143].

Cancer stem cells are associated with therapeutic resistance and decrease in the survival of cancer patients [150]. Moreover, chemoresistance in cancer is associated with various mechanisms, such as mutations, inactivation or elimination of the drug, and overexpression of the therapeutic target [151]. In this sense, in MCF7 cells, leptin also confers resistance to tamoxifen, an anti-estrogen treatment commonly used in cancer patients [150,152]. A possible mechanism was proposed upon the observation that leptin induces an increase in the expression of the estrogen receptor α (ERα) in MCF7 cells treated or not with tamoxifen, suggesting that leptin may confer resistance to this treatment, even in ER-positive cancer cells which are sensitive to tamoxifen [153,154].

## 6. Role of Leptin in EMT in Breast Cancer

One of the first studies that explored the effect of leptin on EMT in mammary cancer cells was carried out by Yan et al. This study showed that in MCF7 cells, leptin stimulation induces a fibroblastoid morphology evidenced by the decrease in the expression of epithelial markers (occludin, E-cadherin) and an increase in mesenchymal markers (fibronectin, N-cadherin, and vimentin) [6]. Also, leptin induces an increase in the expression of β-catenin and, through Akt, induces the phosphorylation at Ser9 in GSK3β, preventing the formation of the destruction complex GSK3β–APC–LKB1–Axin, thus allowing β-catenin to be translocated to the nucleus, form a complex with TLC–LEF, and regulate the expression of cyclin D1 and fibronectin [6].

Leptin induces the overexpression of the EMT markers vimentin and fibronectin and the downregulation of E-cadherin in MCF7 and SK-BR-3 cells, via the activation of the PI3K–Akt signaling cascade and the increased expression of the pyruvate kinase isozyme M2 (PKM2) [155]. Importantly, PKM2 is overexpressed in metastatic tissue compared to non-metastatic breast cancer tissue. Moreover, PKM2 contributes to the maintenance of the cancer stem cells pool via a Wnt–β-catenin-dependent pathway, suggesting an important role of PKM2 in metastasis [155]. Another signaling mechanism involving leptin-induced EMT was observed in MCF7 and SK-BR-3 cells, where leptin promotes IL-8 activation via PI3K–Akt [7]. Similarly, leptin decreases the levels of E-cadherin and induces an increase in the levels of vimentin and Snail in MCF7 cells [149].

In addition, leptin induces the expression of Twist in MCF7, SK-BR-3, and MDA-MB-231 cells [5,6,156] and induces the phosphorylation of STAT3 at Tyr705, allowing STAT3 translocation to the nucleus where it regulates the expression of EMT-associated genes, such as MMP-7 (*MMP7*), MMP-9 (*MMP9*), vimentin (*VIM*), and, importantly, Twist (*TWIST*) [157,158]. Furthermore, leptin induces an increase in Zeb expression due to the formation of the STAT3–G9a complex [159]. G9a is a histone methyltransferase which induces di-methylation of lysine 9 of histone 3 (H3K9Me2), an epigenetic mark associated with transcriptional repression [159]. The STAT3–G9a complex binds to the response elements of the promoter of miRNA-200c, a microRNA repressor of Zeb [159].

WISP2 or CCN5 is a transcriptional repressor that acts as a negative regulator of breast cancer progression [160,161]. In MDA-MB-231 cells transfected with CCN5, an EMT reversion was observed, accompanied by a decrease in the expression of mesenchymal markers such as vimentin and the stem cell marker CD44, and an increase in epithelial markers such as keratin-19 [161]. However, leptin decreases the expression of CCN5 in MCF7 cells, favoring tumor progression by the induction of EMT through a mechanism regulated by the JAK–Akt–STAT pathway [149]. In addition, the decrease of CCN5 and the induction of EMT promote the formation and maintenance of the cancer stem-like cell phenotype [162].

Interestingly, our research group demonstrated that in the non-tumoral epithelial cell line MCF10A, leptin induces a partial-type EMT via a FAK- and ERK-dependent pathway [163]. Partial EMT in these cells conferred both epithelial and mesenchymal characteristics, including the maintenance of cell–cell junctions and collective cell migration [164]. Collective cell migration is part of EMT and contributes to efficient metastasis in some kinds of cancers; it is also necessary for morphogenesis, angiogenesis, and wound healing [165]. Although leptin does not promote changes in E-cadherin expression, it seems to contribute to its re-localization from the plasma membrane to the cytoplasm and to the induction of collective cell migration in MF10A cells [163]. Interestingly, collective cell migration involves cellular clusters with a higher potential to contain circulating tumor cells (CTCs), which can survive in the circulation and metastasize to distal organs [164]. The molecular markers used to classify CTCs are the epithelial markers EpCAM and CK8/18/19 and the mesenchymal markers vimentin and Twist [166,167]. CTCs can be classified by using EMT markers in three groups including epithelial CTCs, biophenotypic epithelial–mesenchymal CTCs, and mesenchymal CTCs phenotypes [167]. In advanced stages of cancer, mesenchymal CTCs are most commonly found, compared with early cancer stages that have epithelial or epithelial–mesenchymal CTC phenotypes [167]. CTCs present a heterogeneity of EMT markers, which supports the idea that EMT generates CTCs, which are key to tumor invasiveness and metastasis and the decreased survival of cancer patients [168].

## 7. Relation between Leptin and Metabolic Reprogramming during EMT in Breast Cancer

Unlike normal cells that use mitochondrial oxidative phosphorylation as the primary source of ATP, tumor cells use aerobic glycolysis [169]. The metabolic reprogramming of tumor cells is called “Warburg effect” and implicates the generation of energy and molecules essential for the synthesis of amino acids, lipids, and proteins necessary for the increased the proliferation, migration, invasion, and survival of tumors cells [170]. Recently, it has been described that metabolic reprogramming is directly related to EMT, particularly, some molecular pathways provide a positive feed-back between EMT and cell metabolism [171]. Metabolic reprogramming is a process partially regulated by the accumulation of lactate, exported by the proton-linked monocarboxylate transporter 4 (MCT4 or SLC16A3), which leads to a decrease in extracellular pH (pHe) [169]. The cellular decrease in pHe is also associated with chemotherapeutic resistance [172], remodeling of the extracellular matrix (ECM), and activation of MMPs [173], key events in the progression of the EMT program.

Several glycolytic enzymes have been associated with EMT program completion. For instance, aldolase A, which catalyzes the reversible conversion of fructose-1,6-bisphosphate to glyceraldehyde 3-phosphate and dihydroxyacetone phosphate, contributes to EMT by promoting the overexpression of N-cadherin and vimentin and decreasing the expression of E-cadherin [169]. Furthermore, PKM2, an enzyme that converts phosphoenolpyruvate to pyruvate, transcriptionally regulates β-catenin by binding to phosphorylated Y333 of β-catenin, allowing its activation [122]. In consequence, PKM2 contributes to the expression of EMT markers such as Snail and vimentin and to the downregulation of E-cadherin [174]. In addition, PKM2 can phosphorylate STAT3, a regulator of EMT markers, such as MMP-2, MMP-9, and Snail, in breast cancer cells [175]. In addition, lactate dehydrogenase, which catalyzes the conversion of lactate to pyruvic acid, is essential for the expression of FAK, VEGF, and MMP2 in MDA-MB-231 breast cancer cell line [169]. On the other hand, the expression of TFs, such as Snail, can induce metabolic reprogramming by inhibiting mitochondrial respiration through the repression of the activity of cytochrome C oxidase [169,176]. In addition, breast cancer epithelial cells undergoing EMT present an increased expression of transporters and enzymes related to aerobic glycolysis and lactate dehydrogenase, and the pentose pathway and the biosynthesis of serine are inhibited during this process [177,178]. Blanquer-Rosselló et al. demonstrated that leptin promotes metabolic reprogramming by favoring mitochondrial biogenesis and energy production processes, essential for the growth and survival of MCF7 breast cancer cells [179].

The association between glucose metabolism and tumor progression has been established; also, lipid metabolism and its association with EMT are currently investigated. Lipid metabolism is an alternative route for energy generation, through lipolysis, fatty acids oxidation, and de novo generation of fatty acids [180]. Tumor cells have a high demand for de novo synthesis of essential fatty acids for the biogenesis of membrane phospholipids due, in part, to their high proliferative rates [180,181]. In this context, leptin induces the rescue of mitochondrial respiration through the use of fatty acids as fuel for the generation of ATP. This metabolic leptin-induced reprogramming confers benefits to tumor cells and a greater aggressiveness to breast cancer cells [182]. On the other hand, lipid β-oxidation is an alternative route for the generation of energy [181]. Wang et al. demonstrated that leptin promotes the oxidation of fatty acids through a JAK–STAT-dependent pathway as well as the self-renewal of BCSC and, consequently, induces chemoresistance of breast cancer cells [183]. Triple-negative breast cancer cell lines express lipoprotein lipase (LPL) and fatty acid synthase, both enzymes participating in lipolysis through the CD36 pathway, which transports fatty acids into the cell [184,185]. In addition, CD36 is associated with the activation of EMT [186].

Therefore, a wide variety of enzymes participate in metabolic reprogramming and are closely related to the progress of the EMT program in breast cancer cells.

## 8. Conclusions

**Figure 3:**
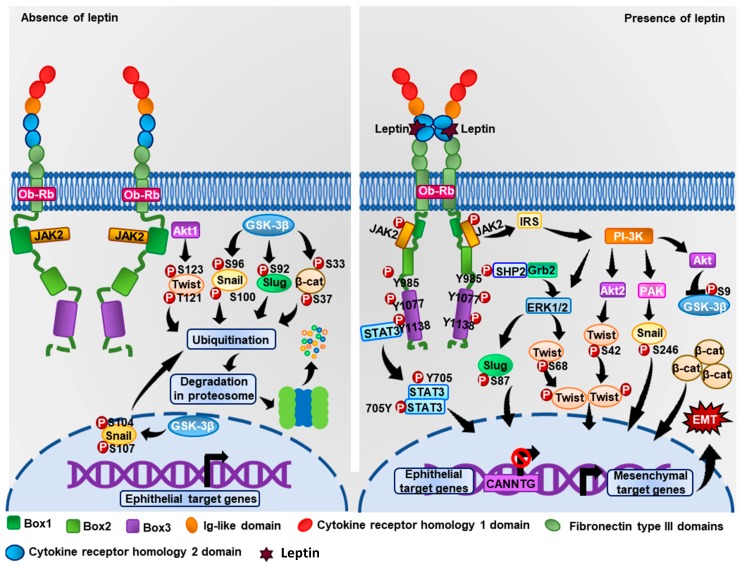
Representative model of leptin-induced EMT signaling. In the absence of leptin, the transcription factors Twist, Snail, Slug, and β-catenin are phosphorylated at specific residues by cytosolic kinases, leading these TFs to their degradation through the proteasomal pathway. When leptin binds to the ObRb receptor, signaling cascades are activated to promote the phosphorylation of EMT-related TFs and induce their translocation from the cytoplasm to the nucleus, where they regulate the expression of EMT-regulators genes.

Currently, breast cancer and obesity are considered a major public health problem worldwide. Several molecules related to obesity have been associated with tumor progression in breast cancer. Leptin promotes diverse biological events associated with essential processes of breast cancer such as EMT. One of the mechanisms by which leptin promotes EMT is through the expression of transcription factors such as Snail, Slug, Zeb, Twist, and β-catenin (Figure 3). These factors repress the epithelial markers while promoting the expression of mesenchymal markers and consequently cell migration, invasion, and metastasis of tumor cells. However, the specific signaling mechanisms by which leptin induces the expression of these TFs and the signaling pathways regulating the EMT markers have not been described completely. Intensive research in the field is now aimed at better understanding the molecular mechanisms that leptin triggers in tumor cells and discovering new molecular targets for therapy in patients with breast cancer and obesity. In conclusion, leptin promotes the progression of breast cancer through the induction of the EMT program, promoting a more aggressive phenotype in breast cancer cells.
